# Hepatitis B virus infection among pregnant women in Ethiopia: a systematic review and Meta-analysis of prevalence studies

**DOI:** 10.1186/s12879-018-3234-2

**Published:** 2018-07-11

**Authors:** Kindie Mitiku Kebede, Dejene Derseh Abateneh, Alemayehu Sayih Belay

**Affiliations:** 1 Department of Public Health, College of Health Sciences, Mizan -Tepi University, PO.box 260, Mizan-Teferi, Ethiopia; 2 Department of Medical Laboratory Sciences, College of Health Sciences, Mizan -Tepi University, Mizan-Teferi, Ethiopia; 3 Department of Nursing, College of Health Sciences, Mizan -Tepi University, Mizan-Teferi, Ethiopia

**Keywords:** Prevalence, Seroprevalence, Hepatitis B, Pregnant women, Ethiopia, Systematic review, And meta-analysis

## Abstract

**Background:**

There are several epidemiological studies available on hepatitis B virus among pregnant women in Ethiopia. These individual studies revealed wide variation over time and across geographical areas. The aim of this systematic review and Meta-analysis is to estimate the overall prevalence of hepatitis B virus infection among pregnant women in Ethiopia.

**Methods:**

A comprehensive search of electronic databases including PubMed, Popline, Lalicus, Ovid, MedNar, African Journal Online (AJOL) and advanced Google Scholar was conducted regardless of publication year from August 30, 2017 to September 25, 2017. The search was updated on January 02, 2018 to minimize time-lag bias. The methodological qualities of included studies were assessed using Joanna Briggs Institute Meta-Analysis of Statistics Assessment and Review Instruments.

**Results:**

Out of 103 studies, 17 studies with a total of 5629 pregnant women were included in the Meta-analysis. The pooled prevalence of hepatitis B virus infection among pregnant women using random-effect model was 4.7%(95% CI 4.0–5.4%). The I^2^ statistics was I^2^ = 37.9%(*p* = 0.0575). Even though significant heterogeneity among studies was not detected, the I^2^ = 37.9% suggests medium heterogeneity. A subgroup Meta-analysis showed that study site, region, mean/median sample size, hepatitis B virus screening methods and methodological quality were not source of heterogeneity (p-difference > 0.05).

**Conclusion:**

This review shows an intermediate level of hepatitis B virus infection among pregnant women in Ethiopia. In addition to the current practice of child vaccination, routine and universal antenatal hepatitis B virus screening program need to be implemented.

**Electronic supplementary material:**

The online version of this article (10.1186/s12879-018-3234-2) contains supplementary material, which is available to authorized users.

## Background

Viral hepatitis is an emerging global health problem. In 2015, an estimated 1.34 million deaths occurred due to viral hepatitis globally. This number is equal to deaths caused by tuberculosis and higher than those deaths caused by human immunodeficiency virus. In the same year, hepatitis B and C viruses (HBV&HCV) alone were responsible for 96% of hepatitis mortality [1]. Untreated hepatitis B and C viral infections can lead to life treating long-term complications such as liver cirrhosis and cancer [2].

Women of childbearing age can potentially transmit HBV to their babies. They transmit an infection to newborn usually during birth or soon after birth following close contact. Newborns who exposed to HBV will have almost 85–90% risk of developing chronic liver diseases [3].

In Ethiopia, the rate of HBV transmission from infected mother to the newborn is not well studied. However, one study revealed that 75% of newborns born from HBV infected women were positive with hepatitis B surface antigen(HBsAg) in 2012 [4].

Hepatitis is becoming an emerging public health concern in Ethiopia. Recent systematic review of all types of viral hepatitis in Ethiopia concluded that the prevalence of HBV among the population is 7.4% [5]. Several HBV epidemiological studies among pregnant women are available in Ethiopia [6–14]. However, the results of these studies showed a wide variation of prevalence ranging from 2.3 to 7.8% [5, 7, 8, 10–14] over time and across geographical areas. Despite the availability of results from each study, there are no nationwide data on the prevalence of HBV infection among pregnant women in Ethiopia which clearly shows the presence of research gaps.

Furthermore, a well organized and synthesized data on viral hepatitis are limited and there was a recommendation to conduct a systematic review. A single systematic review was conducted in Ethiopia [5]. However, in this systematic review, the prevalence of HBV among pregnant women was not estimated.

From the policy perspective, the health burden due to viral hepatitis, in general, are still given less attention in the country’s health system [5, 15]. A recent report showed that not only the general population but also healthcare professional’s awareness of the epidemiology of hepatitis virus is low [10, 15].

Several studies in Ethiopia recommended incorporation of routine antenatal care (ANC) screening program for Hepatitis B [11, 12, 14, 16]. However, regular antenatal screening of pregnant women is not common and compulsory in Ethiopia [17]. The absence of regular HBV screening program could be partly explained by lack of awareness on the overall burden of hepatitis B among pregnant women in Ethiopia by health professionals and policymakers. Therefore, this systematic review was conducted to give a quantitative estimate of the burden of HBV infection among pregnant women as a step to use for a better understanding of its epidemiology in Ethiopia and inform policymakers to take practical action at the policy level.

## Methods

### Setting

Ethiopia is one of the east African countries situated in the horn of Africa. It covers an area of 1.1 k.m^2^ divided in to 9 regions namely Tigray, Afar, Amhara, Oromia, Somali, Benishangul-Gumuz, Southern Nations Nationalities and People Region (SNNPR), Gambella, Harari, and two Administrative states (Addis Ababa city administration and Dire Dawa city administration).

When the first HBV seroprevalence were reported (in 1960s), the population of Ethiopia was 22 million. Currently, the population of Ethiopia is estimated to be > 100 million and it is the second most populous country in Africa next to Nigeria [18].

In Ethiopia, universal vaccination of children against HBV in the form of pentavalent vaccine was introduced in 2007 [19]. The pentavalent vaccine contains HBV vaccine along with Diphteria, pertusis, tetanus, and Haemophilius Influenza B. It is free from charge throughout the expanded program on immunization. However, it is not free from charge for general population including pregnant women. The World Health Organization and the center for disease control and prevention recommend that all health professionals should be vaccinated against HBV before they start the clinical attachments during their stay in the medical school [20, 21]. Accordingly, vaccination of health professionals against HBV in Ethiopia has been started. However, the available studies in Ethiopia showed that vaccination of health professionals against HBV is low ranging from 4 to 28.7% [22–25]. Furthermore, Antenatal screening for HBsAg to all pregnant women and vaccination of their babies at birth has been recommended widely, yet it is not a routine practice in most health settings of Ethiopia.

### Criteria for considering studies for the review

#### Inclusion criteria

##### Design

All observational studies that assessed the prevalence of HBV among pregnant women residing in Ethiopia irrespective of their ANC status were included.

##### Publication status

Only peer-reviewed articles.

##### Language

The articles included in this study were those articles published only in the English language. This is because of feasibility associated with reading and understanding other languages. Furthermore, reporting articles in other languages is uncommon in Ethiopia.

##### Publication or report year

It is preferred that this study will include the past 5 to 10 years for systematic review and Meta-analysis. However, due to the insufficiency of literature, we reviewed all publications irrespective of publication or reporting year.

##### Method of diagnosis

No restriction on methods of diagnosis.

##### Inclusion in the Meta-analysis

We included these primary studies scored ≥60% of The Joanna Briggs Institute (JBI) criteria’s for assessing the quality of primary studies in the meta-analysis.

#### Exclusion criteria

Observational studies including case report and case series were excluded. In case of studies published in more than one report, we excluded the duplicates and select the most comprehensive and up-to-date version. Studies conducted among Ethiopian pregnant women living outside of Ethiopia were excluded. Furthermore, a study conducted by Tsega et al. in 1988 was not included in the Meta analysis. We excluded this study to have a contemporaneous pooled prevalence estimate.

#### Search strategy and information sources

The presence of precursor systematic review and/or protocol on the topic of interest was checked via searching different databases for systematic review. The databases searched include Cochrane database of a systematic review, Joanna Briggs Institute database of a systematic review and implementation reports (JBI-DSRIR), the national health center review and dissemination database, health technology assessment-HTA, the Campbell collaboration library and evidence for policy and practice information (EPPI-centre). The combination of key terms including “HBV”, “hepatitis B”, “pregnant women”, Ethiopia, “systematic review” and protocols were used. The search from the above databases confirmed that there was no systematic review and /or protocol on the topic of interest. Exceptionally, we found one systematic review that reported the prevalence of all types hepatitis viruses among all populations in Ethiopia [5].

After checking antecedent systematic reviews, the actual search was conducted from August 30, 2017-September 25, 2017. The electronic databases searched include PubMed, Popline, Lalicus, Ovid, Med Nar, African Journal Online (AJOL) and advanced Google scholar.

In order to minimize time-lag bias, the searching process was updated on January 02, 2018. The search focused on all published studies with epidemiological data on the prevalence of HBV during pregnancy in Ethiopia. Combinations of key terms such as Hepatitis B, HBV, pregnant women, and Ethiopia were used. These key terms were combined using Boolean operators “AND” and “OR” to narrow the search. The main search strategy conducted in PubMed is shown in the online supplementary file (Additional file 1).

After identifying key relevant articles, their references were also looked into. Similarly, other studies that cited them were viewed online. Two authors (KMK and DDA) conducted the search. An Endnote software version 5 was used to manage references.

##### Study selection

Two reviewers (KMK and DDA) executed the study eligibility assessment independently. The third author (ASB) negotiated any discrepancy between the two authors. The Preferred Reporting Items for Systematic Reviews and Meta-Analyses flow diagram was used to summarize the study selection processes [26].

##### Data extraction and methodological quality

A standardized data extraction tool was adapted from JBI Meta-Analysis of Statistics Assessment and Review Instruments [27]. The two authors (KMK and DDA) extracted the data from included studies independently and checked the data together. From each included studies, information on study area, year of publication, year of sample collection, study design, study region, sample size, time of data collection, and type of HBsAg screening methods was extracted.

A critical appraisal checklist for observational studies (reported prevalence data) adopted from JBI [27] was used to assess the overall methodological quality of included studies. The methodological components assessed include: addressing the target population; appropriateness of participant recruitment; adequacy of sample size; detail description of study subjects; data analysis with sufficient coverage of the identified sample; use of valid methods for identification of the condition; measurement of the condition in a standardize and reliable way for all participants, use of appropriate statistical analysis and adequacy of response rate (Additional file 2). Two authors (KMK and DDA) critically appraise each study independently using a critical appraisal check list adopted from JBI. For any discrepancies during critical appraisal, the third author (ASB) was consulted.

#### Data analysis

Data were analyzed using the *‘*meta*’* packages of the statistical software R (version 3.3.3, The R Foundation for statistical computing, Vienna, Austria). Unadjusted prevalence was recalculated based on crude numerators and denominators provided by individual studies. To minimize the effect of studies with extremely small or extremely large prevalence estimates, the variance of the study-specific prevalence was stabilized with the Freeman-Tukey double arcsine transformation before pooling the data within a random-effects meta-analysis model [28]. Egger’s test served to assess the presence of publication bias [29]. A *p*-value < 0.05 on the Egger test was considered indicative of statistically significant publication bias. Heterogeneity was evaluated by the χ^2^ test on Cochrane’s Q statistic [30], which was quantified by H and I^2^ values. The I^2^ statistic estimates the percentage of total variation across studies due to true between-study differences rather than chance. In general, I^2^ values greater than 60–70% indicate the presence of substantial heterogeneity [31].

The source of variation among studies was assessed with subgroup analysis using grouping variables such as study site, study region, median/mean sample size, HBV screening method and methodological quality.

Furthermore, Cohen’s k coefficient was calculated using SPSS version 21 to determine if there was inter-rater agreement between two reviewers in screening of abstracts and methodological quality assessment [32].

## Results

### Study selection

There were 17 identified peer-reviewed articles that fulfilled the inclusion criteria. The search from PubMed, Popline, Lalicus, Ovid, MedNar, African Journal Online (AJOL) and advanced Google scholar provided a total of 99 studies.

Four additional articles were retrieved from the reference list of the above databases. After removing duplications, 60 studies remain. After reviewing titles and abstracts, 39 articles were discarded because their titles were not related to the topic of interest. Two more studies were discarded because those were conference papers. One study was excluded because of unavailability of the full text.

**Fig. 1:**
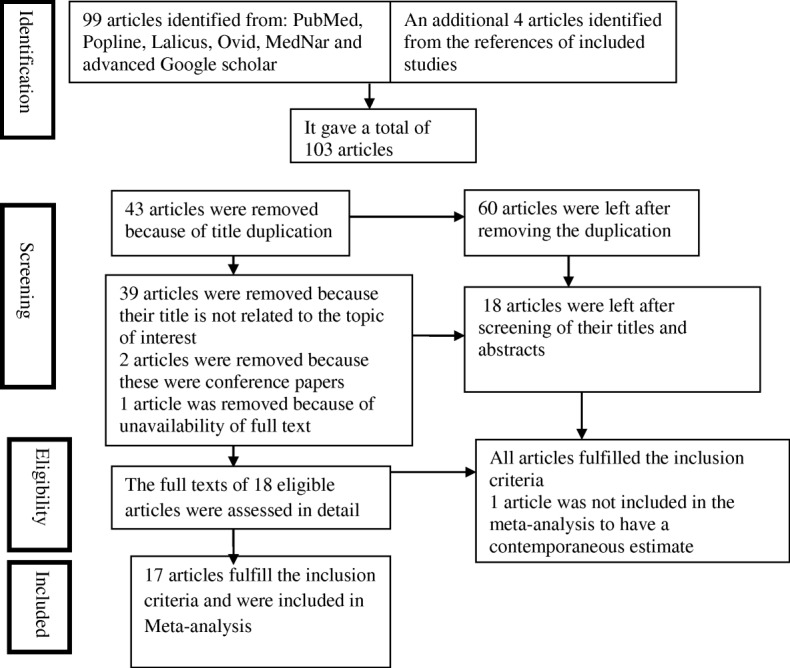
Flow diagram of included and excluded studies

Furthermore, one article was not included in the meta-analysis to have a contemporaneous estimate. There was almost perfect agreement between two reviewers (KMK and DDA) in abstract screening k = 84.9%; *p* < 0.01. The overall selection process of studies was undertaken according to PRISMA Flow Diagram (Fig. 1).

### Characteristics of included studies

All studies fulfilled the eligibility criteria were institutional based cross-sectional studies. Except for one study, the time of data collection for nearly all of the studies was prospective. Majority of the studies (14 studies) used consecutive sampling methods where as 4 studies used random sampling methods. The studies were published from 1988 to 2017 and conducted from 1983 to 2015. Fourteen studies were conducted among antenatal care attendee women where as 4 studies were conducted among pregnant women irrespective of their ANC status. The included studies in the Meta-analysis involved a total of 5629 participants. Nine studies measured the prevalence of hepatitis B virus infection using ELISA and other 9 studies using rapid test. Only two included studies detect hepatitis B e antigen (HBeAg) [33, 34] and assess HBV infectivity among pregnant women. All included studies did not indicate liver health status of HBsAg positive pregnant women. In addition, any of the included studies did not assess the stage of HBV infection (acute or chronic). The studies used test kits from different manufacturers.

The included studies were conducted in various regions of Ethiopia: one in Northern, one in Northeast, three in North West, six in central, one in eastern, four in Southern and two in Southwest Ethiopia. The characteristics of each included study are presented in online supplementary file (Additional file 3).

### Methodological quality of studies

The JBI criteria’s for assessing the quality of primary studies recommend to include primary studies scored ≥60% of methodological checklists in the meta-analysis. Accordingly, we found 6 studies scored≥80% [4, 8, 10–12, 14]; nine studies scored between 70 and 80% [6, 7, 13, 33, 35–39]. Based on the critical review used in this study, there were no studies scored less than 60%. In the majority of the studies (76.5%), the sample size was not adequate [4, 6, 8–14, 16, 36–38]. Importantly, nearly all the studies used valid methods for identification of HBsAg and measured HBsAg in a standardized way (Additional file 4). A substantial inter-rater agreement between reviewers (KMK&DDA) was observed in methodological quality assessment k = 76.9; *p* < 0.01.

### Results of individual studies

According to this review, there is an old study that reported the prevalence of HBV infection among pregnant women at Addis Ababa in 1983 [33]. This study revealed that among 500 pregnant women 5% of them were positive for hepatitis B virus. A study conducted in the Southwest Ethiopia from October 2002–March 2003 revealed that the overall prevalence of hepatitis B among pregnant women is 3.7% with a range of (1.4–6.4%) [40]. Later on, a study conducted in the Northwest Ethiopia from January to March 2004 found a prevalence of 5.3% [6]. A recent study at Hawasa referral hospital in the southern region of Ethiopia found high prevalence (7.8%) of hepatitis B virus infection among pregnant women [7].

Whereas, low prevalence (2.3%) of hepatitis B virus infection among pregnant women was reported in 2012 at Gambo rural hospital in the same region [7, 16].

### Overall pooled prevalence of HBV infection among pregnant women

**Fig. 2:**
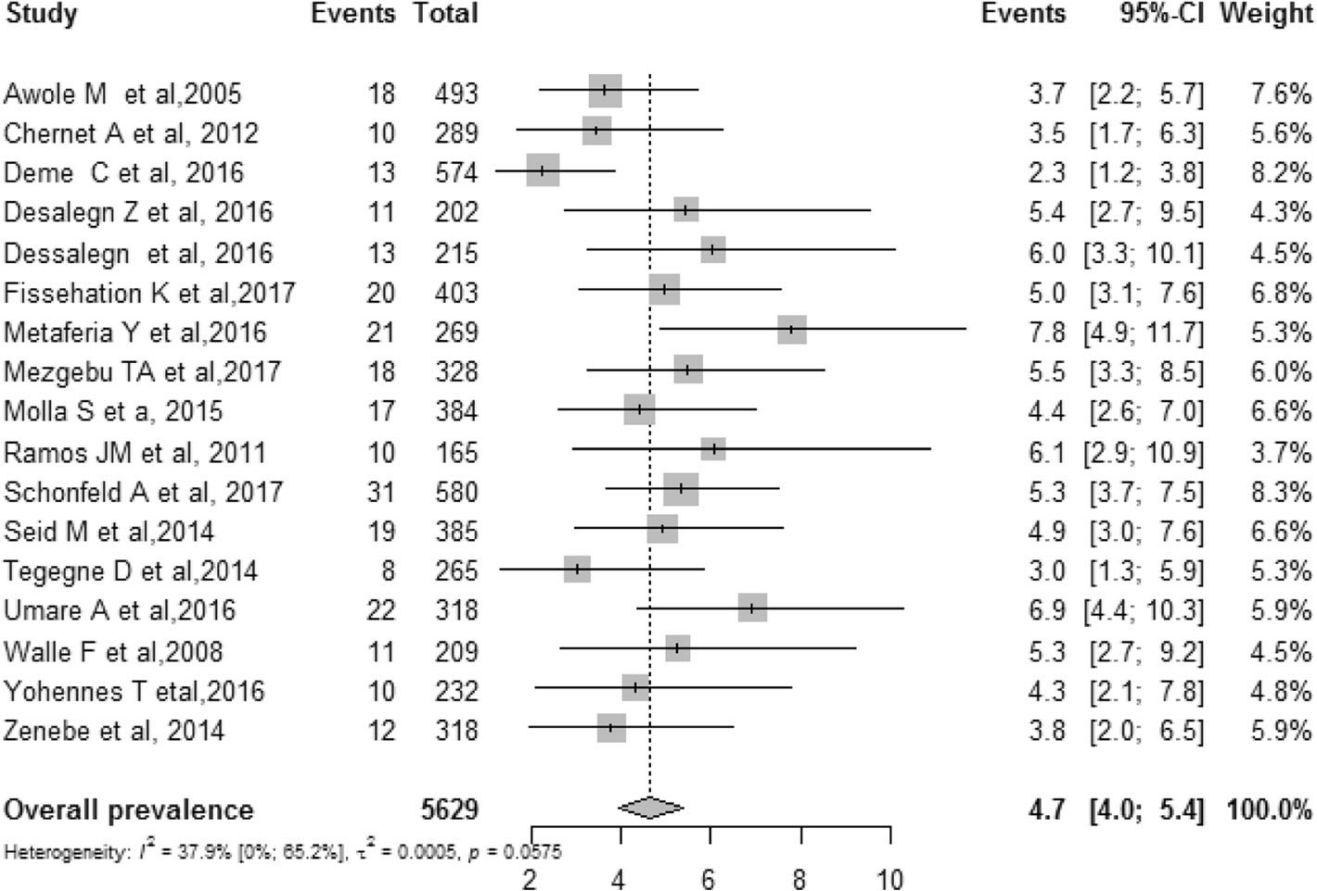
Meta-analysis and forest plot presentation of HBV infection among pregnant women in Ethiopia from 2005 to 2017(based on publication year)

This Meta-analysis included 17 studies where the overall pooled prevalence of HBV infection among pregnant women in Ethiopia was 4.7% (95% CI 4.0–5.4%). The I^2^ statistics for HBV infection among pregnant women was I^2^ = 37.9(*p* = 0.0575). A *p* value of 0.0575 indicates the absence of significant heterogeneity among the included studies (Fig. 2). However, I^2^ = 37.9 suggests medium heterogeneity among studies. We further conducted subgroup Meta-analysis to identify the source of this medium heterogeneity using grouping variables: study site, region, mean/median sample size, HBV screening methods and methodological quality. None of these grouping variables were source of heterogeneity (p-difference > 0.05). The HBV prevalence results for each subgroup analysis are presented as separate online files (Additional files 5, 6, 7, 8, and 9). We also presented The HBV prevalence of all subgroups including assessment of publication bias and assessment of difference between subgroups in Table 1.

**Table 1:** Summary statistics of the meta-analysis prevalence of hepatitis B virus infection among pregnant women in Ethiopia

	Prevalence, % (95%CI)	N studies	N participants	I^2^ (95%CI)	H (95%CI)	p heterogeneity	P Egger test	p difference
Overall	4.7 (4.0–5.4)	17	5629	37.9 (0.0–65.2)	1.3 (1.0–1.7)	0.058	0.076	–
By site								
ANC	4.8 (3.8–5.8)	12	3723	48.6 (0.0–73.6)	1.4 (1.0–2.0)	0.029	0.026	0.664
ANC irrespective	4.5 (3.5–5.5)	5	1906	6.9 (0.0–80.6)	1.0 (1.0–2.3)	0.368	0.939	
By median sample size								
< 318	5.0 (3.9–6.2)	8	1846	22.1 (0.0–64.0)	1.1 (1.0–1.7)	0.253	0.349	0.418
≥ 318	4.5 (3.6–5.5)	9	3783	48.6 (0.0–76.1)	1.4 (1.0–2.0)	0.049	0.148	
By HBV screening tool								
ELISA	5.2 (4.2–6.3)	9	2306	18.8 (0.0–60.5)	1.1 (1.0–1.6)	0.275	0.877	0.122
Rapid	4.2 (3.3–5.2)	8	3323	41.8 (0.0–74.3)	1.3 (1.0–2.0)	0.100	0.320	
Regions								
Central	4.9 (3.9–6.0)	5	1665	0.0 (0.0–74.3)	1.0 (1.0–2.0)	0.519	0.910	0.405
Eastern	6.9 (4.4–10.0)	1	318	NA	NA	NA	NA	
North Eastern	4.9 (3.0–7.3)	1	385	NA	NA	NA	NA	
North West	4.4 (3.1–5.8)	3	911	0.0 (0.0–69.8)	1.0 (1.0–1.8)	0.708	0.534	
Northern	5.5 (3.3–8.2)	1	328	NA	NA	NA	NA	
South West	3.6 (2.4–5.0)	2	782	1.0	0.0	0.928	NA	
Southern	4.8 (2.3–8.0)	4	1240	79.7 (46.0–92.3)	2.2 (1.4–3.6)	0.002	0.198	
Methodological quality								
JBI ≤ 80	4.9 (4.0–5.9)	11	3823	48.4 (0.0–74.2)	1.4 (1.0–2.0)	0.036	0.039	0.405
JBI > 80	4.3 (3.3–5.4)	6	1806	15.7 (0.0–78.6)	1.1 (1.0–2.2)	0.131	0.596	

*CI* confidence interval, *NA* not applicable

### Publication bias

The result of the statistical test for publication bias (Egger’s regression asymmetry test) was statistically non-significant (*P* = 0.108). This result showed no evidence of publication bias. The graphic representation of publication bias with Funnel plot of all included studies is found in online supplementary file (Additional file 10).

## Discussion

The present systematic review and Meta-analysis aimed to estimate the prevalence of HBV infection among pregnant women in Ethiopia. The information obtained from this systematic review and Meta-analysis may improve knowledge on the epidemiology of HBV infection among pregnant women in Ethiopia.

The overall meta-analysis showed that the pooled prevalence of HBV infection in Ethiopia among pregnant women was 4.7%(95% CI 4.0–5.4%). This finding is close to WHO endemic definition of HBV infection(5–7%) [41] and prevalence of chronic hepatitis B in Ethiopia estimated by Aparna Schweitzer et al.*..* (6.03%) [42]. However, this finding is lower than the prevalence of HBV infection among general populations in Ethiopia (7.4%) [5]. This may indicate the risk of HBV infection among pregnant women is lower than the risk of the general population. Alternatively, the high prevalence of HBV infection in the general population may be because of high mean age among the general population than the mean age of pregnant women. Moghaddasifar I et al also postulated that, the low prevalence of HBV infection among Iranian pregnant women(1.2%) is due to their lower mean age [43].

The available systematic reviews and Meta-analysis in Africa for instance in Nigeria, Cameron and Ghana showed a high prevalence of HBV infection among pregnant women ranging from 9.8 to 14.1% [34, 44, 45]. The findings from Nigeria, Cameron and Ghana are higher than that of the estimates from this Meta analysis. This difference could be attributed to the real difference in the prevalence of HBV infection among the general population. The prevalence of HBV infection among the general population in Nigeria, Cameron and Ghana were 13.6, 11.8 and 12.3% respectively that is higher than Ethiopian estimate (7.4%) [5].

Furthermore, the difference in the risk of contracting HBV infection, socio-economic, environmental and behavioral risk factors may contribute to this difference.

In the contrary, the prevalence of HBV infection among pregnant women in Ethiopia is higher than estimation from several studies in European and American countries such as Denmark (0.26%) [46], Brazil(1%) [47], Spain (0.1%) [48], Guatemala (0.22) [49], Southeastern Turkey(1.74%) [50] and Northern Turkey (2.1%) [51]. The low prevalence of HBV infection among pregnant women in Europe and America may show the effect of higher socio-economic status, level of hygiene and vaccination coverage.

In this Meta-analysis, we couldn’t detect publication bias using eggers regression test. Lack of significant publication bias in the regression test might be the absence of significant dispersion in the sample among the included studies. It is well documented that lack of significant dispersion and reasonable sample size may cause non-significant eggers regression test [52]. Borenstein M et al. [52] argued even in the presence of significant dispersion and reasonable numbers of studies, regression test has lower power. Therefore, the failure to find evidence of publication bias using regression test should not lead to a false sense of assurance.

The intermediate level of HBV infection among pregnant women in this systematic review may indicate policymakers and programmers to implement routine and universal HBV screening program for all pregnant women.

Several other studies in Ethiopia [8, 10–14, 16], a meta-analysis in Iran [43] and studies in other countries [50, 53–55] also recommended the establishment of antenatal HBV screening program. Universal screening program enables women to access HBV treatment. Furthermore, it may increase child vaccination coverage. For instance, in Denmark after implementation of universal screenings for hepatitis B, vaccination coverage raise to 96% [46].

The finding from this systematic review needs to be interpreted cautiously because all studies included in this systematic review and Meta-analysis were institutional based studies which covered a portion of pregnant women in Ethiopia. These women might be relatively healthy, educated and economically empowered so that their risk of HBV infection is minimal. Furthermore, the included studies used different HBsAg screening tools. Some of the screening tools (Linear Chemicals, Joaquim Costa, and Barcelona, Spain) cannot detect less than 1 ng/mL of HBsAg in specimens. As result, false negative results may occur if the quantity of HBsAg present in the specimen < 1 ng/mL. In rare cases, HBsAg tests do not detect certain HBV mutant strains. Mutated HBsAg may not be detected by the available HBsAg screening tools (Linear chemicals. Joaquim Costa, Barcelona, Spain) as a result false negative results could be occurred. All these limitations may underestimate the true pooled prevalence of HBV infection among pregnant women in Ethiopia.

### Limitations and strengths of the study

Most of the studies included were conducted in more recent years. As a result, the prevalence estimates in this Meta-analysis is likely to show the current situation of HBV infection among pregnant women in Ethiopia.

The full text of one article was not accessed. Efforts were made to access this article from the primary and coauthors through email and phone. However, positive response was not acquired. As a result, to some extent, this review might not be free from full publication bias.

Finally, in this review, we included articles published only in the English language. Thus, the introduction of language bias is expected. However, publication in other languages in Ethiopia is uncommon. For this reason, the introduction of language bias is minimal.

## Conclusion

Given the above-mentioned limitations, our systematic review and Meta-analysis confirmed the intermediate level of HBV infection among pregnant women in Ethiopia. In addition to behavioral education and communication, we recommend implementation of universal and routine regular antenatal screening program for HBV for all pregnant women in Ethiopia.

## Additional files

Additional file 1: Searching steps for PubMed. (DOCX 11 kb)

Additional file 2: JBI Critical Appraisal Checklist for Studies Reporting Prevalence Data. (DOCX 12 kb)

Additional file 3: The prevalence of HBV infection among pregnant women in different regions of Ethiopia from 1988 to 2017. (XLSX 20 kb)

Additional file 4: Quality assessment of included studies using the Joanna Briggs Institute criteria’s for assessing quality of primary studies. (DOCX 14 kb)

Additional file 5: Sub-group meta-analysis by study site among pregnant women in Ethiopia. (DOCX 1401 kb)

Additional file 6: Sub-group meta-analysis by study region among pregnant women in Ethiopia. (DOCX 2330 kb)

Additional file 7: Sub-group meta-analysis by median sample size among pregnant women in Ethiopia. (DOCX 1435 kb)

Additional file 8: Sub-group meta-analysis by HBV screening tools among pregnant women in Ethiopia. (DOCX 1515 kb)

Additional file 9: Sub-group meta-analysis by JBI methodological quality among pregnant women in Ethiopia. (DOCX 1467 kb)

Additional file 10: Graphic representation of publication bias using funnel plots of all included studies. (DOCX 1318 kb)

## Abbreviations

ANC: Antenatal care
ELISA: Enzyme-linked immunosorbent assay
HBeAg: Hepatitis B Virus e antigen
HBsAg: Hepatitis B Virus surface antigen
HBV: Hepatitis B Virus
HCV: Hepatitis C Virus
HIV: Human Immunodeficiency virus
JBI: Joanna Briggs Institute
WHO: World health organization
