# Supplementary material for: Hepatitis B virus infection among pregnant women in Ethiopia: a systematic review and Meta-analysis of prevalence studies
Source: BMC Infect Dis. 2018 Jul 11;18:322. doi: 10.1186/s12879-018-3234-2 (PMC6042274; doi:10.1186/s12879-018-3234-2)
Supplement: Supplementary file 1 — Searching steps for PubMed. (DOCX 11 kb) [file 12879_2018_3234_MOESM1_ESM.docx]

Searching steps for PubMed

1. Prevalence
2. seroprevalence
3. “Hepatitis B”
4. “HBV”
5. “pregnant women”
6. Ethiopia
7. 1OR 2
8. 3 OR 4
9. 7 AND 8 AND 5 AND 6
