# Supplementary material for: Hepatitis B virus infection among pregnant women in Ethiopia: a systematic review and Meta-analysis of prevalence studies
Source: BMC Infect Dis. 2018 Jul 11;18:322. doi: 10.1186/s12879-018-3234-2 (PMC6042274; doi:10.1186/s12879-018-3234-2)
Supplement: Supplementary file 4 — Quality assessment of included studies using the Joanna Briggs Institute criteria’s for assessing quality of primary studies. (DOCX 14 kb) [file 12879_2018_3234_MOESM4_ESM.docx]

Table 1: quality assessment of included studies using the Joanna Briggs Institute criteria’s for assessing quality of primary studies

| Study | Q1 | Q2 | Q3 | Q4 | Q5 | Q6 | Q7 | Q8 | Q9 | Score % |
| --- | --- | --- | --- | --- | --- | --- | --- | --- | --- | --- |
| Zenebe Y etal,2014 | Y | Y | N | Y | Y | Y | Y | Y | Y | 88.89 |
| Molla S etal,2015 | Y | Y | N | Y | Y | Y | Y | Y | Y | 88.9 |
| Dessalegn etal, 2016 | Y | Y | N | N | Y | Y | Y | Y | Y | 77.78 |
| Yohenn Z etal,2016 | Y | Y | N | Y | Y | Y | Y | Y | Y | 88.89 |
| Chernet A etal, 2012 | Y | Y | N | Y | Y | Y | Y | Y | Y | 88.89 |
| Umare A,2016 | Y | Y | N | Y | Y | Y | Y | Y | Y | 88.89 |
| Mezgebu TA,2017 | Y | U | N | Y | Y | Y | Y | Y | Y | 77.78 |
| Fissehation K,2017 | Y | U | N | Y | Y | Y | Y | Y | Y | 77.78 |
| Walle F etal,2008 | Y | U | N | Y | Y | Y | Y | Y | Y | 77.78 |
| Seid M etal,2014 | Y | Y | N | Y | Y | Y | N | Y | Y | 77.78 |
| Tegegne D etal,2014 | Y | Y | N | Y | Y | Y | Y | Y | Y | 88.89 |
| Scho¨ nfeld A etal,2017 | Y | N | Y | Y | Y | Y | Y | Y | N | 77.78 |
| Desalegn Z etal, 2016 | Y | N | N | N | Y | Y | Y | Y | Y | 66.67 |
| Metaferia Y etal,2016 | Y | N | N | N | Y | Y | Y | Y | Y | 66.67 |
| Deme C etal ,2016 | Y | Y | Y | Y | U | Y | U | Y | Y | 77.78 |
| Awole M etal,2005 | U | U | Y | N | Y | Y | Y | Y | Y | 66.67 |
| Tsega E etal, 1988 | Y | N | Y | N | Y | Y | Y | Y | Y | 77.78 |
| Ramos JM etal,2011 | Y | Y | N | Y | Y | Y | U | Y | Y | 77.78 |
| Note:  Y - Yes, N - No, U - Unclear  Q1= Was the sample frame appropriate to address the target population?  Q2= Were study participants sampled in an appropriate way?  Q3= Was the sample size adequate?  Q4= Were the study subjects and the setting described in detail?  Q5= Was the data analysis conducted with sufficient coverage of the identified sample?  Q6= Were valid methods used for the identification of the condition?  Q7= Was the condition measured in a standard, reliable way for all participants?  Q8= Was there appropriate statistical analysis?  Q9= Was the response rate adequate, and if not, was the low response rate managed appropriately? | | | | | | | | | |  |
