# Supplementary figures and images for: Hepatitis B virus infection among pregnant women in Ethiopia: a systematic review and Meta-analysis of prevalence studies
Source: BMC Infect Dis. 2018 Jul 11;18:322. doi: 10.1186/s12879-018-3234-2 (PMC6042274; doi:10.1186/s12879-018-3234-2)

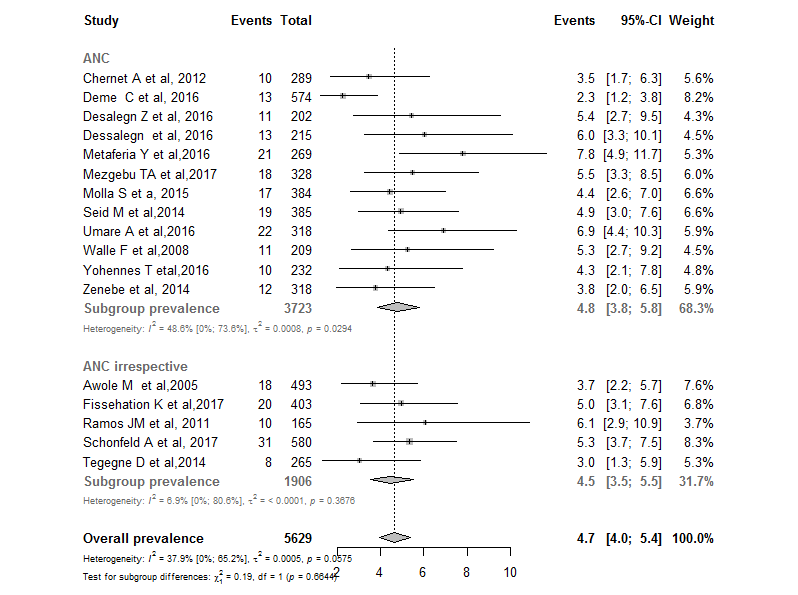

Supplement: Supplementary file 5 — Sub-group meta-analysis by study site among pregnant women in Ethiopia. (DOCX 1401 kb) [file 12879_2018_3234_MOESM5_ESM.docx]

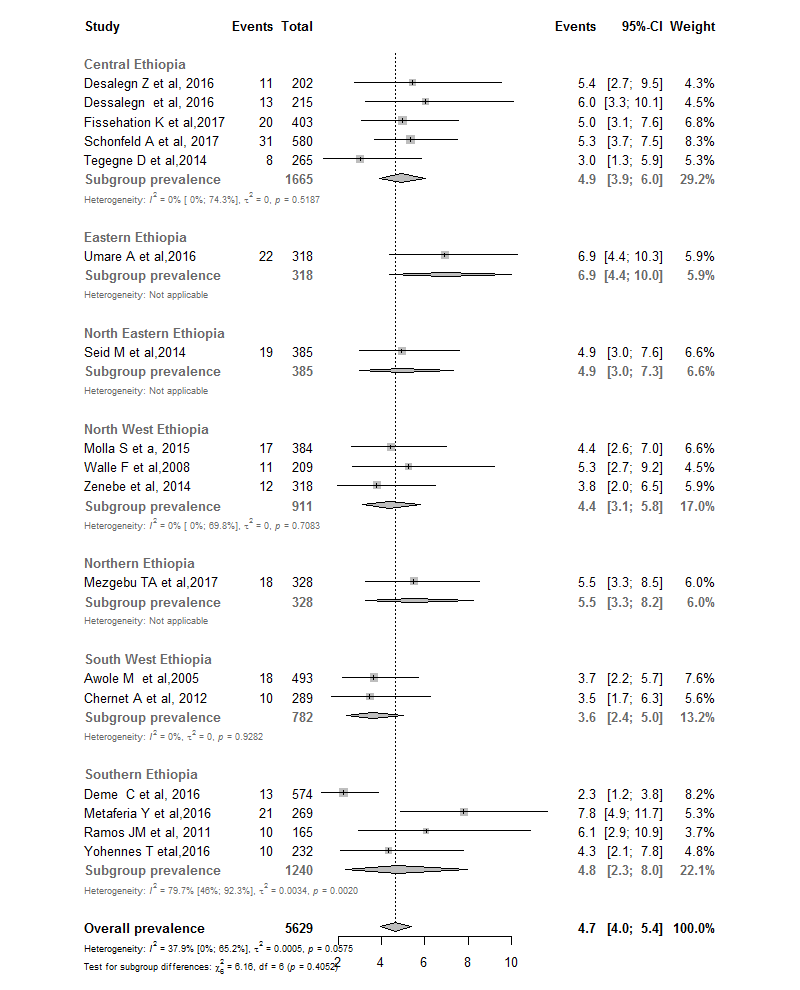

Supplement: Supplementary file 6 — Sub-group meta-analysis by study region among pregnant women in Ethiopia. (DOCX 2330 kb) [file 12879_2018_3234_MOESM6_ESM.docx]

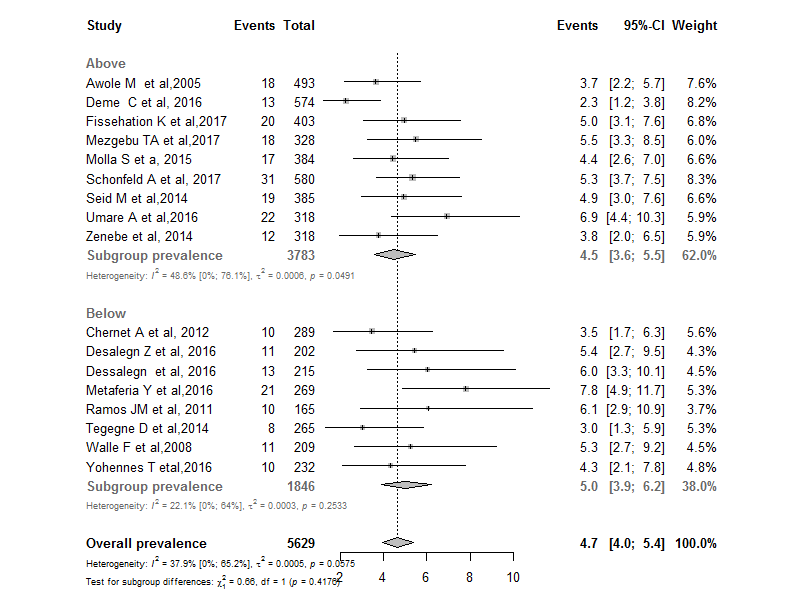

Supplement: Supplementary file 7 — Sub-group meta-analysis by median sample size among pregnant women in Ethiopia. (DOCX 1435 kb) [file 12879_2018_3234_MOESM7_ESM.docx]

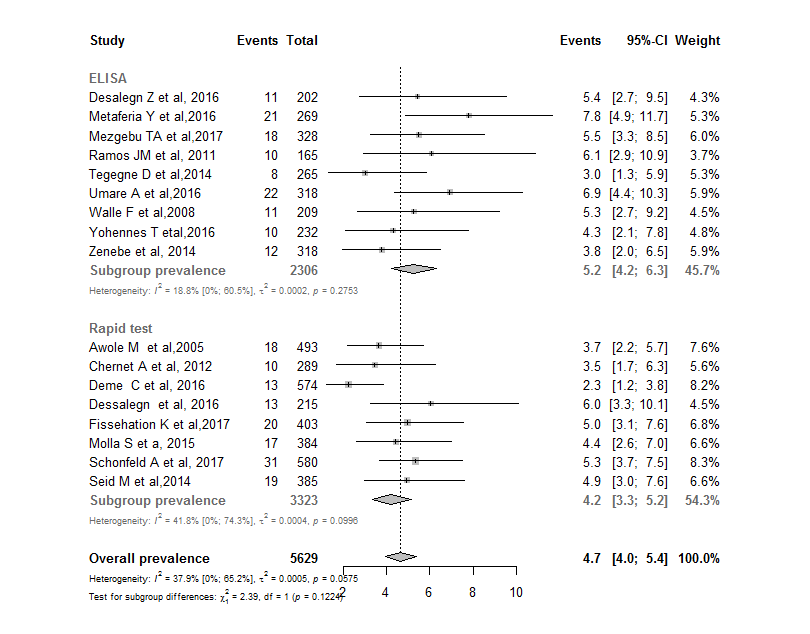

Supplement: Supplementary file 8 — Sub-group meta-analysis by HBV screening tools among pregnant women in Ethiopia. (DOCX 1515 kb) [file 12879_2018_3234_MOESM8_ESM.docx]

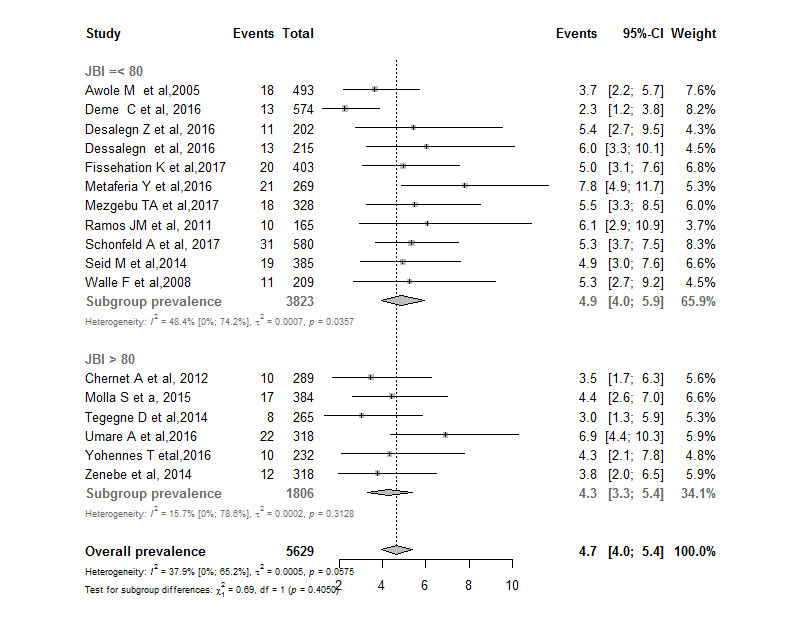

Supplement: Supplementary file 9 — Sub-group meta-analysis by JBI methodological quality among pregnant women in Ethiopia. (DOCX 1467 kb) [file 12879_2018_3234_MOESM9_ESM.docx]

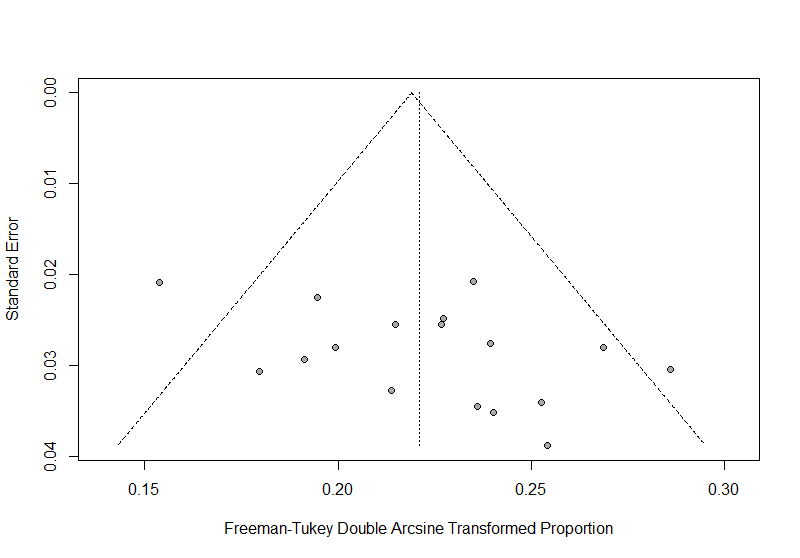

Supplement: Supplementary file 10 — Graphic representation of publication bias using funnel plots of all included studies. (DOCX 1318 kb) [file 12879_2018_3234_MOESM10_ESM.docx]
